# Development of plasma functionalized polypropylene wound dressing for betaine hydrochloride controlled drug delivery on diabetic wounds

**DOI:** 10.1038/s41598-021-89105-7

**Published:** 2021-05-05

**Authors:** Leila Zahedi, Pedram Ghourchi Beigi, Mojtaba Shafiee, Fatemeh Zare, Hamed Mahdikia, Majid Abdouss, Mohammad-Amin Abdollahifar, Babak Shokri

**Affiliations:** 1 Laser and Plasma Research Institute, Shahid Beheshti University, G.C, Velenjak Avenue, Tehran, Iran; 2 Department of Biology and Anatomical Sciences, School of Medicine, Shahid Beheshti University of Medical Sciences, Evin, Tehran, 1985717443 Iran; 3 Department of Chemistry, Amirkabir University of Technology, 424 Hafez Avenue, P. O. Box 15875-4413, Tehran, Iran; 4 Department of Physics, Shahid Beheshti University, G.C, Velenjak Avenue, Tehran, Iran

**Keywords:** Biopolymers in vivo, Biochemistry, Biomaterials, Biopolymers, Polymerization mechanisms, Drug delivery, Drug delivery, Chemical physics, Plasma physics

## Abstract

Diabetes Mellitus is one of the most worrying issues among illnesses, and its chronic subsequences almost refer to inflammations and infections. The loading and local release of antioxidants to wounds may decrease inflammations. However, the low wettability of PolyPropylene (PP) restricts the drug from loading. So, to increase the adhesion of PP for loading an optimum amount of Betaine Hydrochloride (BET), plasma has been applied in two steps of functionalization and polymerization, which has been confirmed with FE-SEM, ATR-FTIR, and EDX. The new chemistry of the surface led to almost 80% of BET loaded. The drug-releasing ratio studied by HPLC approved the presence of a PEG-like layer, which was coated by polymerization of tetraglyme. To evaluate the wound healing potential of the application of PP meshes treated by plasma, 72 Wistar rats were subdivided into four groups. The skin injury site was removed and underwent biomechanical tests, stereological analysis, and RNA extraction. The results showed a significant improvement in the polymerized scaffold containing BET for skin injury. The present study suggests that the use of a modified PP mesh can induce tissue regeneration and accelerate wound healing at the skin injury site.

## Introduction

Diabetes Mellitus (DM) is defined as a metabolic illness due to the deficiency in production, function, and the mixture of insulin leading to an increase in blood glucose^1^. Diabetes comes with microvascular and macrovascular^2^. This illness has two very important side effects (symptoms): peripheral neuropathy (PN)^3^ and deficiency in healing wounds^2^. Peripheral neuropathy is the most important issue for physicians all over the world since it is often diagnosed very late or after the diabetic wound is created on the foot (the most advanced form of PN). The risk of cutting the lower organ in DM is more than 20 times compared to that in the healthy people. People suffering from diabetes may encounter disorders in wound healing^3^. Besides, a number of studies have pointed out the importance of the sufficient capability of veins and the reproduction of vessels in the improvement of tissues and its absence in treatment of the diabetic wound^4^. The process of normal wound treatment is the result of the balance between synthesis and tissue damage. One stage of this process is the demolition of the extracellular matrix (ECM) which does not lead to healing of chronic wounds like diabetics^5^. No final cure has been yet found for diabetes, and the existing treatments merely suffice the appropriate management of the wound^3^. Therefore, it is time to introduce new strategies, for example, systems of drug delivery.

The drug delivery system (DDS) is defined as a formula or an instrument that enables a medical substance to enter the body and improve its efficiency and safety by means of the rate of control, the time, and the place of releasing drugs. Synthetic release control in DDS is controlled by the device itself. Therefore, external factors such as gastrointestinal stimulation and absorption rate do not play an important role in the release of an active substance. One of the most important benefits of controlled drug delivery is the ability to maintain the optimal drug concentration at the target site, leading to more effective treatment, increased immunity to prevent the concentration of toxic drugs, possibility of using drugs with a narrow treatment window and short half-life, and comfort improvement for patients^6^. Various controlled-release kinetics of the drug, controlled by plasma processing, were studied using plasma processing, including electrospun mats^7–9^, surgical mesh^10^, films^11–13^, microparticles^14,15^, microneedles^16,17^, tablets^18,19^, and ceramic materials^20,21^. Depending on the medical application, different release kinetics are required. For example, rapid release of the drug may be desirable for pain relief or sedatives, while the stable and significantly slower release is necessary for hormonal and anti-cancer treatments^14^. Polymer carriers for drug delivery are among the new drug transmitters. Vulnerable implants for stable and controlled delivery of drugs are the scaffolds through which skin tissue engineering overcomes the limitations of several processes of wound improvements such as autografts, allografts, and xenografts^22^. As we know, tissue engineering success has a great role in the efficient embedding of cells, biological scaffolds, and cell signals like growth factors. The construction of a 3D scaffold is one of the major stages in all the techniques of tissue engineering, which imitate the extracellular matrix that acts as a physical support for performing the connection of the original cell to the formation of the next tissue^23^.

As mentioned above, polymers are new drug transmitters, Polypropylene (PP) meshes have been used in medical practice specializing in different surgeries since long years ago^10^. The powerful mechanical features of PP have been repeatedly proven during these years. However, the response that the host soft tissue gives is different and depends on various factors such as chronic inflammation and the tissue connected to the condensed fiber^24^. Yet, this polymer despite its advantages has its own disadvantages. PP potentially includes such traits as less adhesion and less hydrophilicity which leads to a disorder in the direct molecule loading on polymer^10^. Infections created by using surgery meshes are of the other disadvantages of using this biocompatibility polymer^25^. In this research, after creating a wound on each rat, a proper dose of Gentamicin was injected. Therefore, to improve the surface of the polymer, plasma processes were used as a set of operations that could be both economical and safe^10^.

Plasma treatment is a process that is quick, clean, and unstable. By choosing the suitable gas, plasma can add a certain element or a functional group on the surface^26^. Polypropylene polymer along with oxygen gas can be used in plasma treatment and can be oxidized into such groups as hydroxyl, carboxyl, hydrophilic carbonyl^25^. PP surface shows resistance to most chemical solvents leading the adhesion of the biomolecules to be weakened. In this case, cold plasma gets its own importance so much^26^. The plasma treatment attraction among other techniques of ordinary surface improvement such as graft polymer and wet chemistry is that plasma has the ability to activate the only surface characteristics, up to the depth of a few nanometers, with no impact on the volume features of the matter^27^. Plasma processing can be performed under pulse^10,28–31^, or periodic wave discharge^9,11,12,32^, The presence of active ions and photons with a high level of energy in plasma during periodic waveforms can cause the destruction of drug molecules. Therefore, the choice of pulsed wave state can be justified by reducing the probability of chemical changes in the drug that are included in the processed material^29^.

BET is a chemical substance that comes from the betaine existing in marine invertebrates, wheat, alfalfa, etc. However, its most important resource is sugar beet. Betaine (glycine betaine/ methyl glycine-tri-N,N,N/TMG) is an essential osmolyte that, as the main resource of methyl groups in mammals, has a role in one-carbon metabolism. A lot of Betaine urinary is often observed in diabetic people and patients with kidney insufficiency. This urination can be a prediction for side effects of heart and vascular problems in patients who suffer from vascular illness, and the density of dimethylglycine in blood plasma, the product of betaine metabolism, can be considered as a strong sign of the possible future impacts. These predictions about diabetic people are very accurate^33^. Due to the nature of the betaine osmolyte, its shortage endangers the regulator of the cell volume. Also, an increase in betaine urination and an increase in repelling osmolyte sorbitol of the kidney have a direct relationship with diabetic illness^34^. This the reason why we used BET in this research.

In this study, we developed plasma treated PP meshes to improve wound healing in type 1 diabetic rats. We hypothesized that the combined applications of BET and PP meshes with plasma treatment, to an experimental model of diabetic skin wound, may accelerate angiogenesis and cell proliferation, and decrease inflammation.

## Experimental

### Materials

Surgical meshes woven of PP, packed in sterilized packages in 30*30 cm, manufactured by the company of *COUSIN BIOTECH* in France were chosen for this research. In this study, meshes are cut and prepared into a circle in a diameter of 2 cm.

The drug needed for fixing or regulating the betaine lost due to diabetes is betaine hydrochloride (153/61 g mol^−1^) made by *Sigma Aldrich*. This drug shows the solution in water in 1/86 mg mL^−1^ and is used to be mixed with PP mesh.

Tetraethylene glycol dimethyl ether (tetraglyme, *Sigma Aldrich*) CH3O(CH2CH2O)4CH3 was used as the precursor in plasma polymerization.

Commonly Phosphate Buffer saline with pH 7/4 because of the similarity and the ion density and pH in the human body is used in biological researches. This buffer of Na2HPO4, KCl, NaCl, and KH2PO4 (*Sigma Aldrich*) was distilled by twice-distilled water. In addition, NaOH (*Sigma Aldrich*) was used for regulating the amount of its pH.

Streptozotocin with the abbreviation of STZ (*Santa of Cruz Biotechnology*) (265/2 g mol^−1^) is widely used in diabetic induction in an animal model. This drug causes destruction in the beta cells of the pancreas where, in this case, hyperglycemia and non-excretion of insulin are observed in their plasma.

In order to prevent infection on the first day of creating a wound on the mice, Gentamicin was injected.

### In vitro section

#### Plasma surface functionalization

Functionalization of the surface by plasma treatment was done by a plasma cleaner manufactured by the *Satia* Co*. *In this treatment, the samples, under the diverse conditions, and the pressure of 200 mTorr were irradiated with plasma. In order to find the best time and the power for activation of the surface of the surgical PP meshes, powers of 50 W and 70 W and the treatment time of 10, 15, 20, and 30 min were studied. During the experiment, samples were put on the electrode and the flow of the oxygen gas inside the chamber was fixed on 5 sccm. In addition, in order to minimize the effect of aging on the treated samples, all analysis and the next stages of the tetraglyme introduced research were carried out immediately after the activation of the surface of the meshes.

#### Drug loading of PP meshes

PP meshes were cut into circles of 2 cm in diameter and about 0.0115 g in weight. A medical solution of betaine hydrochloride with a various density of 100, 500, 1000, 5000 ppm was produced. First, the sample was put into the solution which was kept inside of the incubator for 24 h with continuous vibration of 160 rpm at a temperature of 20 °C. Then, the samples were dried inside an oven for 24 h at a temperature of 37 °C. This process was repeated for each sample four times. Besides, to get the optimum mode, several tests were done for each group.

#### Plasma polymerization layers

Low pressure radio-frequency plasma (13.56 MHz) was applied for the polymerization of polyethylene glycol (PEG) by bubbling argón—a carrier gas—through liquid to the plasma chamber. In this stage, plasma in the device was used in the pulse mode. The fixed traits of the treatment were: duty cycle of 20%, 4 ms, Power of 70 W, and Pressure of 200 mTorr. Moreover, this treatment was studied by introducing Argon, as a diluent gas, and changing the time of the process. These parameters are shown in (Table 1). Before the main treatment, a pretreatment was done for the activation of the surface in the continuous mode. The features of this treatment were as follows: pressure: 230 mTorr, Argon gas Flow: 10 sccm, Time Duration: 30 s, and Power: 70 W.

**Table 1:** Parameters of the plasma polymerization process.

Name	Carrier gas flow (sccm)	Diluent gas flow (sccm)	Time (min)
PEG1	10	–	15
PEG2	2	8	15
PEG3	10	–	30
PEG4	2	8	30

#### In vitro release analysis

In order to obtain an adequate rate of delivered betaine hydrochloride (BET), a High-Performance Liquid Chromatography (HPLC) device (*Agilent 1200 Technologies* Co.) was employed, and the samples were immersed into a solution of 20 mL of PBS. To get a graph of drug delivery, the process of sampling from the buffer lasted 10 days. A piece of aluminum foil was put into the solution to make the mesh heavy enough to be completely immersed and get in touch with the drug and buffer. The above process was repeated for each sample at least four times and compared with the control sample as a reference.

#### Surface topography test (FE-SEM, EDX)

The topography of the plasma-treated and untreated PP meshes was surveyed by Field-Emission Scanning Electron Microscope (FESEM) of *MIRA3TESCAN-XMU*. Observations were made under the voltage of 15 kV. This device was also equipped with second-generation microanalysis of the Scattered Energy of X-ray (EDX) in such a way that it provided the possibility of the recognition of the smallest phases. According to the possibility of regulating the pressure inside the device compartment with high resolution, the percentage of the chemical elements on the surfaces of the treated and untreated meshes was surveyed and analyzed^35^.

#### Attenuated total reflection (ATR-FTIR)

The Infrared absorption spectra were obtained by a Fourier Transform Infrared (FTIR) spectrophotometer equipped with an Attenuated Total Reflection (ATR) sample holder (*Bruker Equinox55 instrument Germany* in the range of 600–4000 cm^−1^). For each spectrum obtained, a total of 40 scans were accumulated at 4 cm^−1^ resolution.

#### High-performance liquid chromatography method (analysis) (HPLC)

In order to find and set up a methodology to efficiently determine the betaine in different chemical compositions or various feed ingredients, a number of studies have been accurately carried out^36^. In these studies, the breakdown of BET has been conducted by means of the C8 column in the HPLC device. This column is rarely found. Therefore, after a lot of trial and error we finally drew a conclusion that using C18 column could be a new method although it has not been mentioned in the previous studies. This method is as follows:

The density of BET for the standard and test sample was displayed by HPLC (*Agilent system, USA*). A stable phase column (C18 column, 4.6 150 mm) with a flow rate of 1.0 mL min^−1^ was sustained at room temperature. The mobile phase solvent was phosphate-buffered saline (0.01 mol L^−1^, pH 4). The samples were observed and quantified at 200 nm using a UV–Vis detector. The injection volume was 10 μL. The mobile phase was filtered through a 0.22 mm polytetrafluoroethylene (PTFE) filter (Millipore, USA). The retention time of BET was roughly 1.41 min.

#### In vivo studies

##### Ethical approval

The study was conducted in strict accordance with the recommendations, and the protocol was approved by the Institutional Research Ethics Committee of school of Medicine—Shahid Beheshti University of Medical Sciences (IR.SBMU.MSP.REC.1399.234), and followed the replacement, reduction, and refinement principles for experimental animals. All experiments were performed in accordance with the relevant guidelines and regulations. All methods were carried out in compliance with the ARRIVE Guidelines 2.0.

##### Experimental animals

In total, 72 male Wistar rats with a mean weight of 200 g ± 20 g and 8-week old were obtained from the laboratory animal center of Shahid Beheshti University of Medical Sciences, Tehran, Iran. One wound (skin injury) was inflicted in each of the 72 Wistar rats. Type 1 diabetes mellitus (T1DM) was induced in the 65 male rats which were randomly divided into four groups: Group I: untreated control (healthy) (C), Group II: untreated control (diabetic) (D), Group III: Functionalized scaffold containing BET (diabetic) (D + F + B), Group IV: Functionalized and polymerized scaffold containing BET (diabetic) (D + F + B + P). Each group of six rats was housed under standard conditions (room temperature and 12:12 h light–dark schedule) and had free access to water and food (four rats in each cage). On days 7, 15, and 28 we carried out stereo-logical and quantitative Real-Time Polymerase Chain Reaction (RT-PCR) assessments.

#### Induction of T1DM

The rats from groups 2 to 4 received an one-time injection of intraperitoneal streptozotocin (STZ, 40 mg kg^−1^, body weight) to induce T1DM^37^. One week following the STZ injection, blood samples were taken from the veins of rats’ tails in order to analyze the blood glucose levels (Biomine, Rightesttm GM300, Biomine Corporation, Switzerland). Rats with blood glucose levels greater than 250 mg dl^−1^ were considered to be type 1 diabetic. In our study, the rats' blood glucose levels were recorded every 2 weeks until the end of the research. Tissue samples were conducted 30 days after the STZ injection.

#### Surgery

After the injection of STZ in diabetic rats, the animals were anesthetized by ketamine hydrochloride (Trittau, Germany) (50 mg kg^−1^ body weight) injected intramuscularly along with diazepam (50 mg kg^−1^ body weight). Subsequently, the fur on the back of the rats was shaved and cleaned with alcohol and povidone-iodine (Behvazan, Iran). The rats underwent general anesthesia. We used a skin biopsy punch to inflict one full-thickness 2 cm round excisions skin injury on the backs of the rats under hygienic conditions.

#### Analysis of TNF-α, IL-α and KGFs expression by real-time PCR

The total RNA samples were extracted and treated with DNase I (Roche, Basel, Switzerland) to remove genomic DNA contamination. Complementary DNA (cDNA) was synthesized in a total volume of 20 μL using a commercial kit (Fermentas, Lithuania) at 42 °C for 60 min according to the manufacturer's instructions. The RT-PCR (TaqMan) was used for quantification of relative gene expression according to QuantiTect SYBR Green RT‐PCR kit Takara Bio Inc, (Japan). All studied forward and reverse primer pairs were designed according to the Primer 3 Plus software (http://www.bioinformatics.nl/primer3plus)^38^. In exon–exon junction way to distinguish between cDNA and genomic DNA. Previously, the PCR primers were tested by the Primer‐Blast tool at the site, www.ncbi.nlm.nih.gov/tools/primer‐blast (Table 2)^39^.

**Table 2:** The PCR primers.

Genes (accession number)	Primer sequences
KGF	F = GGCAATCAAAGGGGTGGA R = CCTCCGCTGTGTGTCCATTTA
IL1-α	F = TGTGTTGCTGAAGGAGATTCCG R = AAGCTGCGGATGTGAAGTAGT
TNF-α	F = ATGGGCTCCCTCTCATCAGT R = GCTTGGTGGTTTGCTACGAC

#### Tissue preparation

Histological evaluation was performed 30 days after the STZ injection. Tissue samples were fixed in 10% formalin for 1 week and then embedded in paraffin blocks and cut longitudinally into 5 μm and 25 μm thick sections with a microtome. Ten sections of each animal were selected in a systematic random manner. For the microscopic descriptive analysis of each group, slides were stained by hematoxylin and eosin (H&E).

#### Histological and stereological analyzes

##### Number of fibroblasts, neutrophil, and basal cells

The number of inflammatory and skin cells at the healed site was estimated by the optical dissector method. Microcator was applied to measure the Z-axis movement of the microscope stage. In order to avoid biased counting, we selected only the cell nuclei which were completely inside the counting frame and did not cross the exclusion line. The numerical density of cells is estimated by the following equation:

1 $$N=\frac{\sum Q}{\sum P\times h\times \frac{a}{f}}\times \frac{t}{BA},$$

where “*ΣQ*” is the number of the cells' nuclei, “*h*” is the height of the dissector, “*a/f*” is the frame area, “*ΣP*” is the total number of counting frames in all fields, “*t*” is the real section thickness measured using the microcator and *BA* is the section thickness.

##### The volume of epidermis, dermis

The volume of the epidermis, dermis at healed tissue was estimated by Cavalieri's principle equation:

2 $$V=\sum P\times a/p\times t,$$

where *t* is the distance between the sampled sections, *ΣP* is estimated by the point-counting method, and *a/p* is the area associated with each point projected on the skin tissue.

##### Estimation of the length of the vessels

The length of the vessels was measured, using the following formula:

3 $${L}_{v}=\frac{2\Sigma Q}{\sum P\times a/f}.$$

#### Estimation of wound closure area

**Figure 1:**
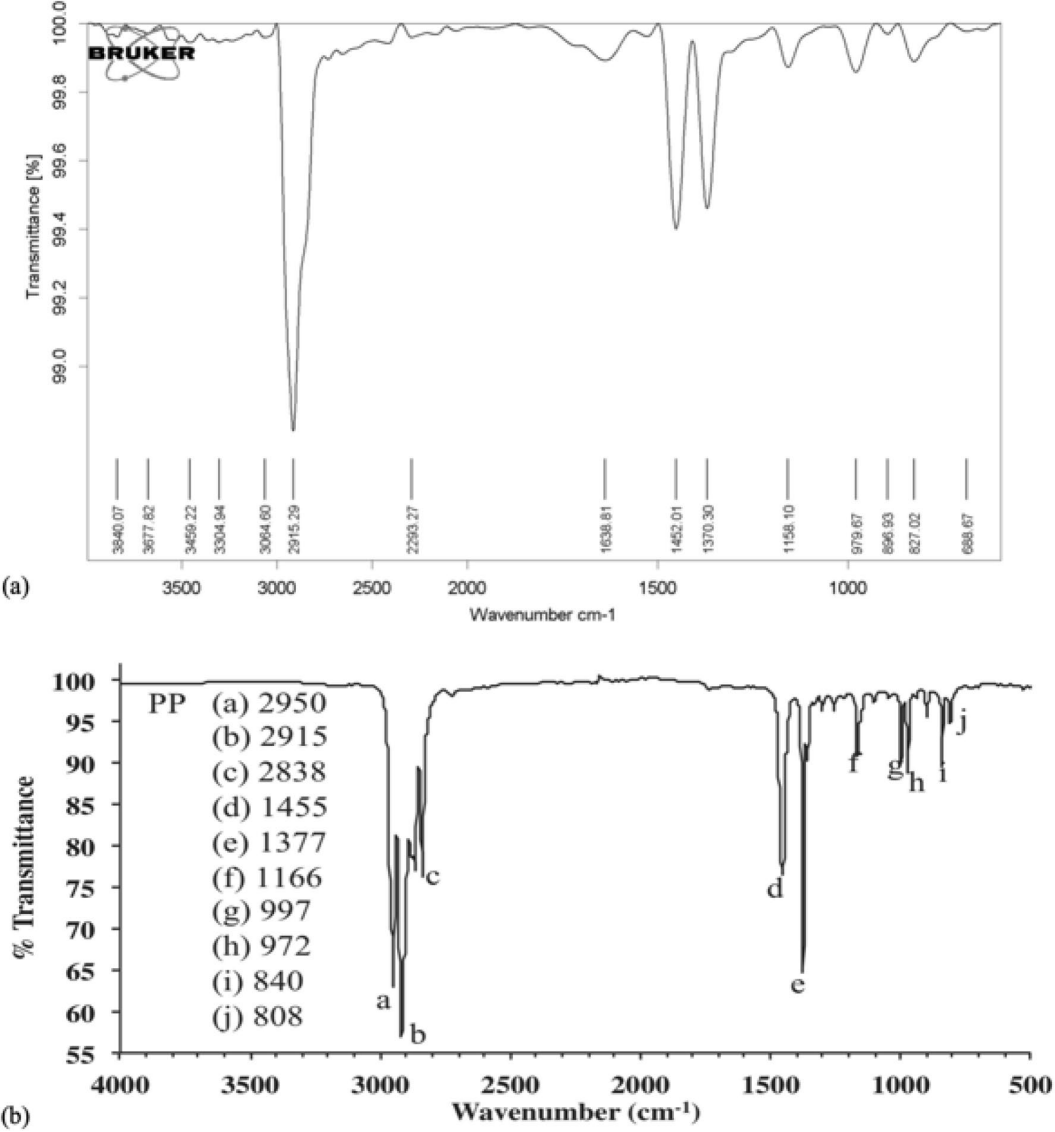
ATR FT-IR spectra of (**a**) plasma treated PP mesh with oxygen gas, (**b**) Control PP mesh.

In the present study, by measuring the wound area, the degree of wound closure area and wound contraction was evaluated using ImageJ software (NIH, version 1.52a). The wound images were taken by a digital camera on days 7, 15, and 28 in each studied group (n = 6 in each subgroup). A standard ruler was set at the level of the wound at the same magnification, and the photos were prepared and imported into ImageJ software and the mean pixel cm^−2^ was determined to calculate accurately the associated surface area of each wound. Then, the surface area of each wound was represented as cm^2^ (Fig. 1).

#### Tensiometer test (tensile strength test) (biomechanical examination)

On days 7, 15, and 28 after surgery, rats were euthanized by inhalation of chloroform in a closed box. After careful dissection from the underlying deep fascia, a uniform sample (2 mm × 20 mm strips) was extracted to cross each wound and normal adjacent skin, by a double-blade cutting standard instrument. The sutures were removed and the specimens were frozen at − 80 °C. On the day of the biomechanical test, specimens were retrieved from the freezer, allowed to melt at room temperature before they were measured by a digital caliper for thickness and length. During the test, specimens were kept moistened. Specimens were mounted in a material testing machine (*Zwick, BZ2.5/TH1S, Germany*) that used two clamps with a rough surface, with the wound in the middle of the free surface^40,41^. Hence, the failure and complete load-deformation curve could be recorded. Indeed, from the curve, bending stiffness (MPa), maximum force (N), stress high load (N mm^−2^), and energy absorption (J) were extracted^42^.

### Statistical analysis

All the results were reported as mean ± standard deviation (SD). We used one-way for parametric and two-way for non-parametric analysis of variance (ANOVA) and the least significant difference (LSD) tests to analyze the stereological and qRT-PCR assessments. A p-value of < 0.05 was considered to be statistically significant.

## Results

### Influence of plasma treatment on the surface chemistry of PP mesh (ATR-FTIR)

In ATR-FTIR analysis, the spectra of plasma treated sample with oxygen gas showed extra peaks on 3459 cm^−1^ and 1638 cm^−1^, in difference with the spectra of control sample. These peaks are related to the OH, and vibrational C=O groups. The apparition of such functional groups as hydroxyl and carbonyl on the surface of the wound dressing suggests that there be an increase in the rate of the surface hydrophilicity and wettability that leads to the improvement of surface adhesion (Fig. 1)^43,44^.

### Influence of plasma treatment on the surface chemistry of PP mesh (EDX)

**Figure 2:**
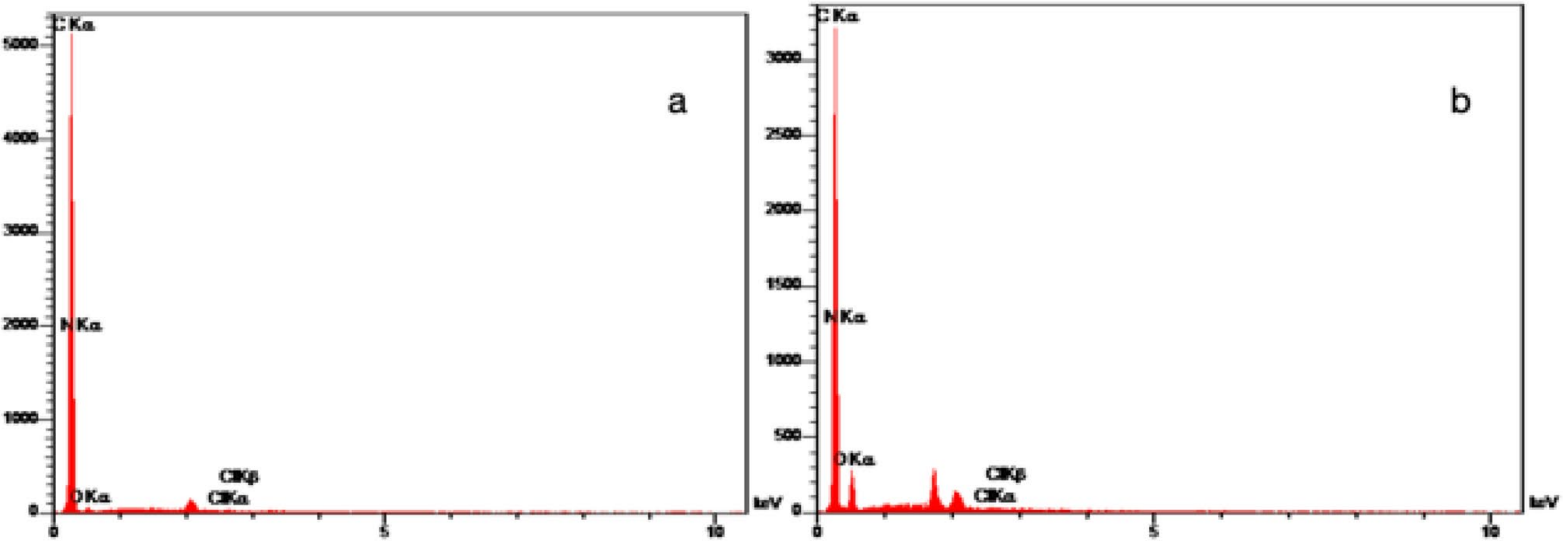
The EDX spectrum of (**a**) F + B and (**b**) F + B + P PP meshes.

Figure 2 displays the element analysis of PP Control and modified meshes using EDX. The untreated PP contains only carbon (C) atoms^25^. Plasma-activated PP mesh with loaded BET illustrates (C) carbon atoms with the addition of (O) oxygen and (N) nitrogen atoms. Figure 2b shows the presence of carbon (C), oxygen (O), and nitrogen (N) atoms. Nitrogen atoms confirm the presence of BET on the surface of the mesh (Table 3). Also, BET has chlorine atoms (Cl) in its structure which is shown in both Fig. 2a,b.

**Table 3:** Average values of atomic percent of C, O, N and Cl as obtained by EDX technique for Control, F + B and F + B + P PP meshes.

	C (%)	N (%)	O (%)	Cl (%)
Control	100	0	0	0
F + B	89.16	6.71	4.07	0.06
F + B + P	72.03	10.51	17.32	0.14

### Influence of plasma treatment on the topographical assets (FESEM)

**Figure 3:**
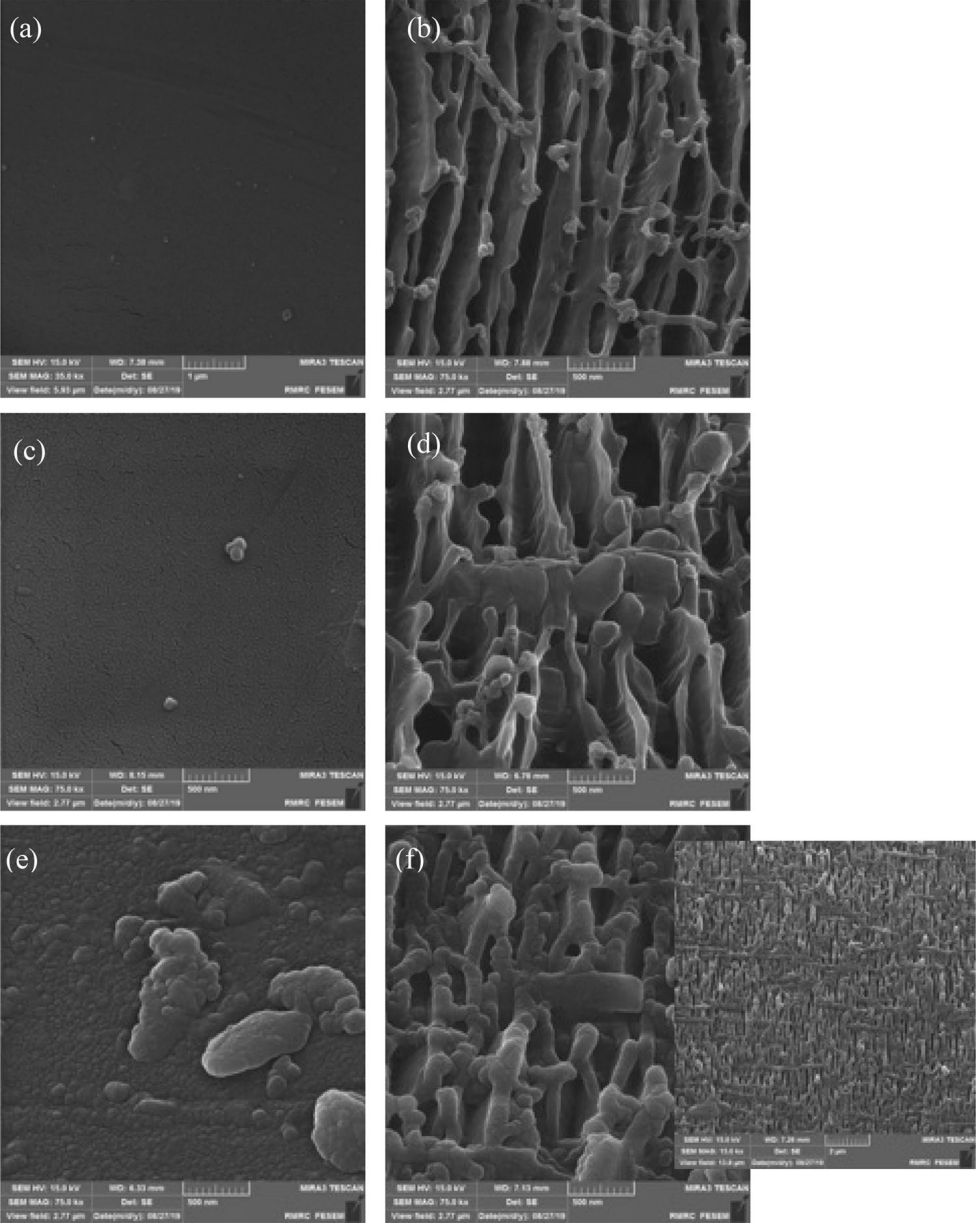
FE-SEM micrographs at $\times$ 75,000 magnification of (**a**) control PP mesh, (**b**) plasma-treated PP mesh, (**c**) BET loaded control PP mesh, (**d**) BET loaded plasma-treated PP mesh, (**e**) BET loaded plasma-polymerized PP mesh, (**f**) BET loaded plasma-treated and plasma polymerized PP mesh, with the corresponding magnification at × 15,000.

Figure 3a,b are related to the untreated and the plasma-treated sample in 15 min with a power of 70 W, respectively, which illustrate the increase of roughness of PP mesh surface. Figure 3c,d refer to untreated and plasma-treated BET loaded sample, respectively. In Fig. 3d, the large molecule of BET seemingly is connected to the surface. Figure 3e refers to the BET loaded untreated PP surface on which the PEG-like layer has been polymerized and the giant molecules of BET can be seen. Figure 3f illustrates the polymerized sample on which the drug is loaded and indicates on the creation of a layer of PEG polymer on the surface in comparison with the plasma-treated drug-loaded sample. Also, at 15,000× magnification, the creation of the networks of polymerized PEG is proved (Fig. 3f)^45^.

### Influence of plasma treatment on the loading and realising of betaine (HPLC)

Drug loading is done by submerging the treated PP mesh into a solution of BET and the samples were rotated inside the incubator at the temperature of 20 °C with 160 rounds per minute for 24 h. Then, immediately after that, it was dried inside the oven at the temperature of 37 °C for 24 h. Drug release is carried out by putting PP mesh containing BET into the PBS buffer solution with 7.4 pH. For this purpose, samples were taken from this solution at different hours in 240 h and were analyzed with the use of HPLC, under the conditions discussed in “High-performance liquid chromatography method (analysis) (HPLC)”.

**Figure 4:**
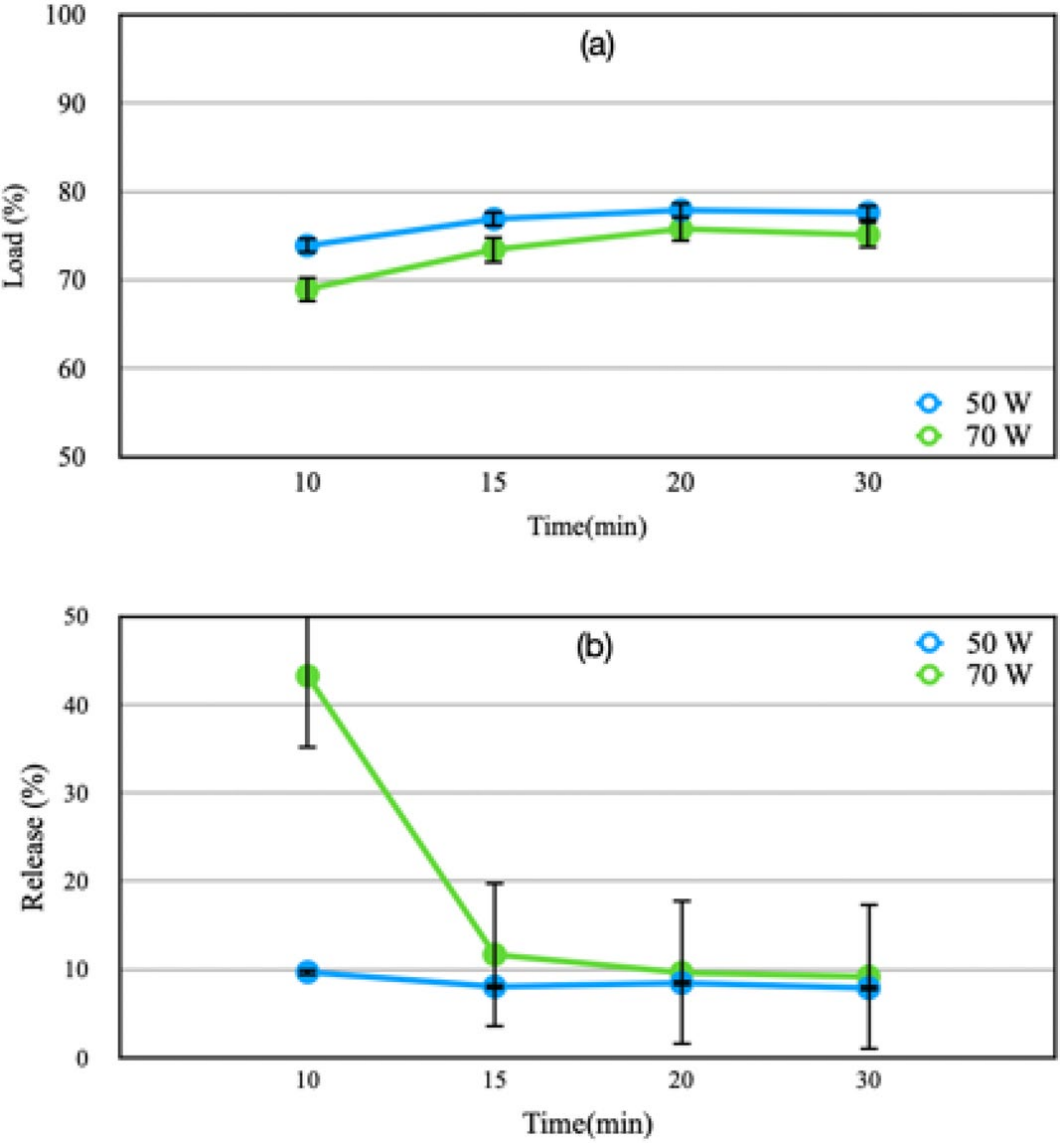
(**a**) BET loading percentage and (**b**) BET releasing percentage of the PP meshes as a function of F time.

According to Fig. 4a, the amount of the drug loaded on the plasma-treated sample at a power of 50 W is a bit more than the amount of the drug-loaded on the plasma-treated sample at a power of 70 W. However, surveying the release of the drug loaded, it is revealed that the plamsa-treated sample at 50-W power manifests the release value less than 10% compared to the drug released from the plasma-treated sample at 70 W power (Fig. 4b). However, in spite of the amount of more drug loaded and with respect to the amount of drug released, the sample treated at 70 W power is chosen as the best sample.

**Figure 5:**
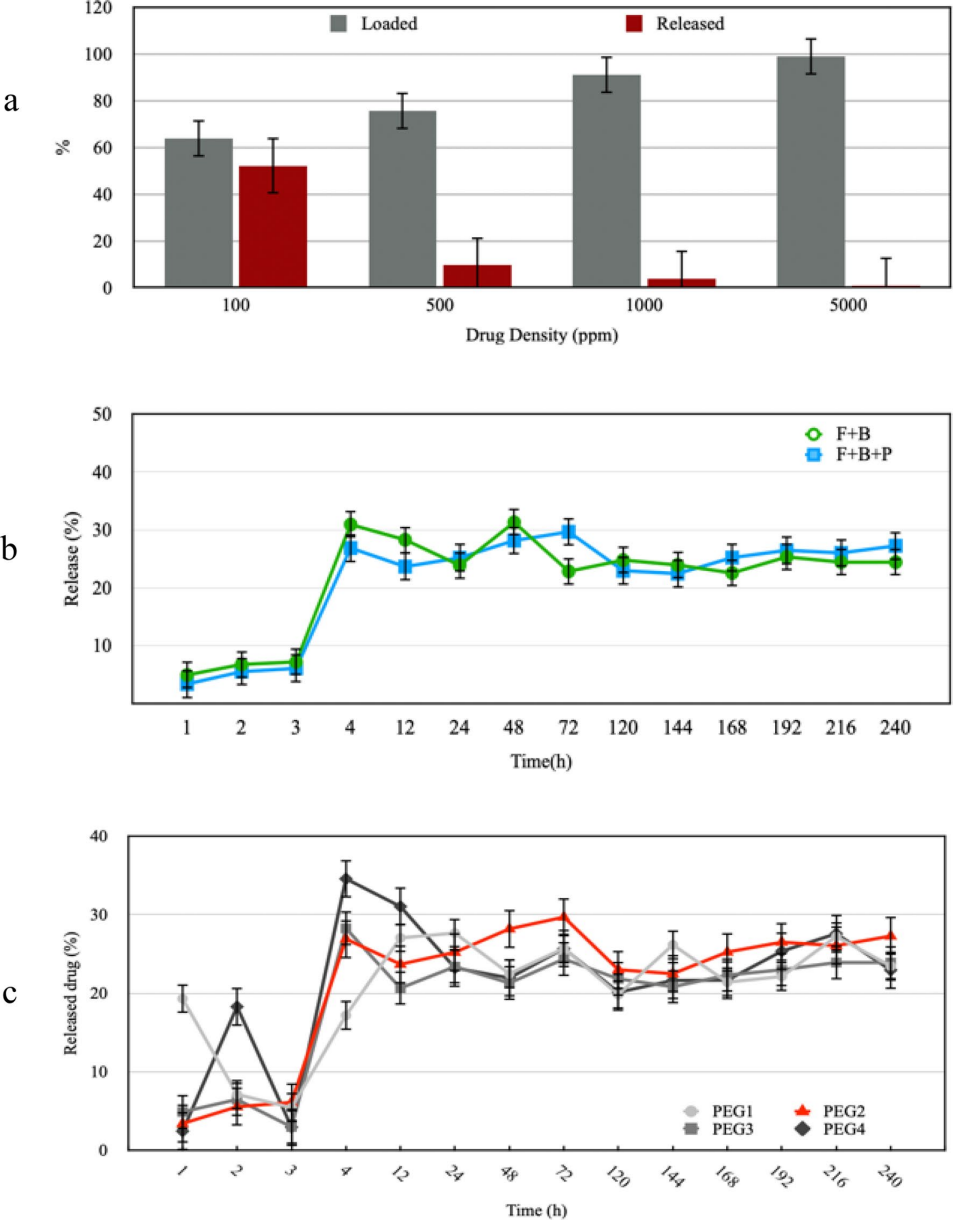
(**a**) Comparison of the effect of the density of the original solution of BET on the percentage of drug loading and releasing. (**b**) Comparison of the drug releasing percentage of the polymerized surface of F + B PP meshes. (**c**) Comparison of the drug releasing percentage of the surface of F + B and F + B + P PP meshes.

Figure 5a shows the rate of the drug loaded and drug released of the surface of the plasma-treated sample at 70-W power in 10 days. The highest amount of drug loaded (around 99%) is related to the sample submerged into a solution of BET with a density of 5000 ppm, while the rate of the released drug is less than 1%. The optimum case is for the sample submerged into a drug solution with a density of 500 ppm with a loading of about 70% and a release of about 10%.

In order to obtain the optimum conditions of plasma polymerization, samples were studied according to Table 1. With regard to the procedure of drug release in different hours (Fig. 5b), we can conclude that the PEG2 sample is in the closest manner to zero-order controlled release^46^. As a result, plasma polymerization is performed under the condition that Argon gas has been used as a carrier gas and a diluting gas with a flow of 2 sccm and 8 sccm, respectively. This process lasted for 15 min.

The efficiency rate of the drug loading for the sample activated with 15-min plasma treatment at 70-W power and the submersion into the drug solution of BET with a density of 500 ppm approximately equals 86%. Figure 5c reveals an early burst release in the fourth hour for both samples, while its rate for the functionalized sample and the functionalized-polymerized sample is about 31% and 27%, respectively. This burst release may be for the sake of drug release which is loosely absorbed in the surface^47,48^.

### Wound closure area

**Figure 6:**
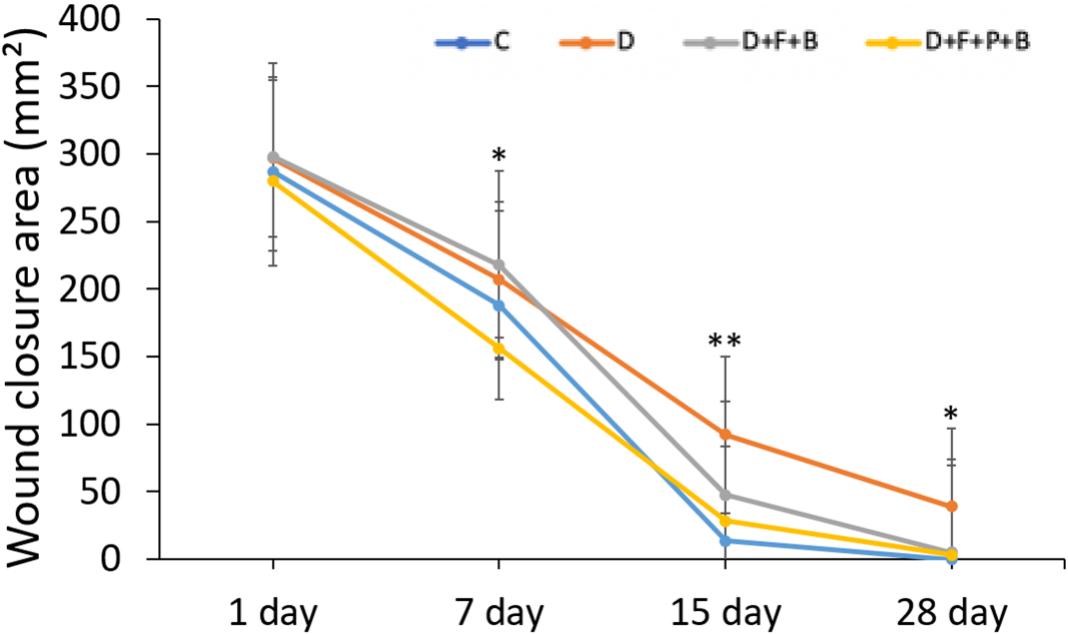
The wound closure area (mm^2^) at days 1, 7, 15 and 28 post injury in the wound area of the different groups are shown. Mean ± SD of the wound closure area in the study groups as compared by the ANOVA and LSD; (*P < 0.05, **P < 0.01 and ***P < 0.001). *ANOVA* analysis of variance; C; D; D + F + B; D + F + B + P, *LSD* least significant difference.

**Figure 7:**
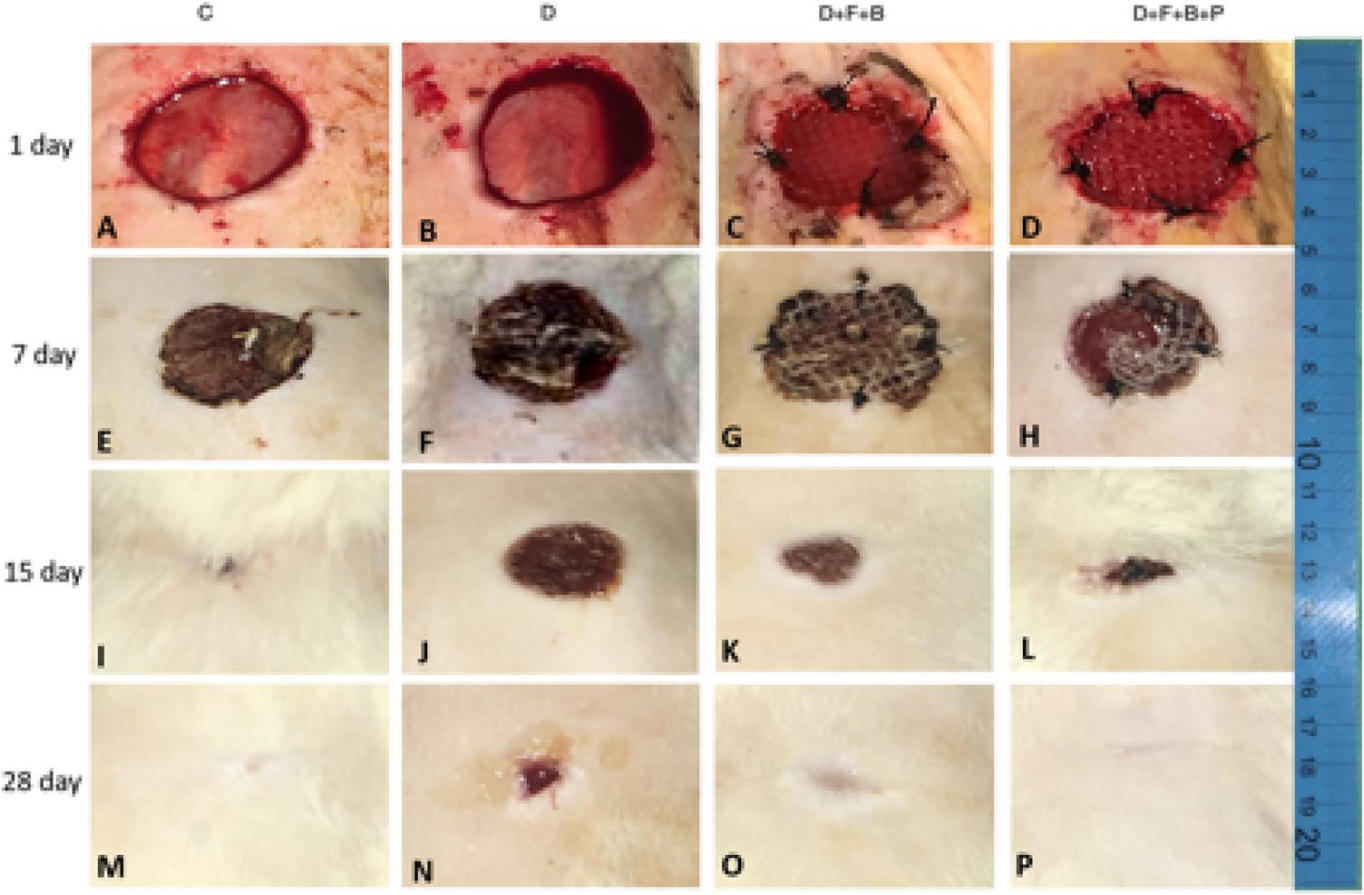
Quantification of wound closure rate. Gross digital camera images illustrating wound closure area in Control, diabetic, D + F + B, and D + F + B + P at the different time points. The pictures show that repairing process at the 1st, 7th, 15th and 28th day in the study groups.

**Figure 8:**
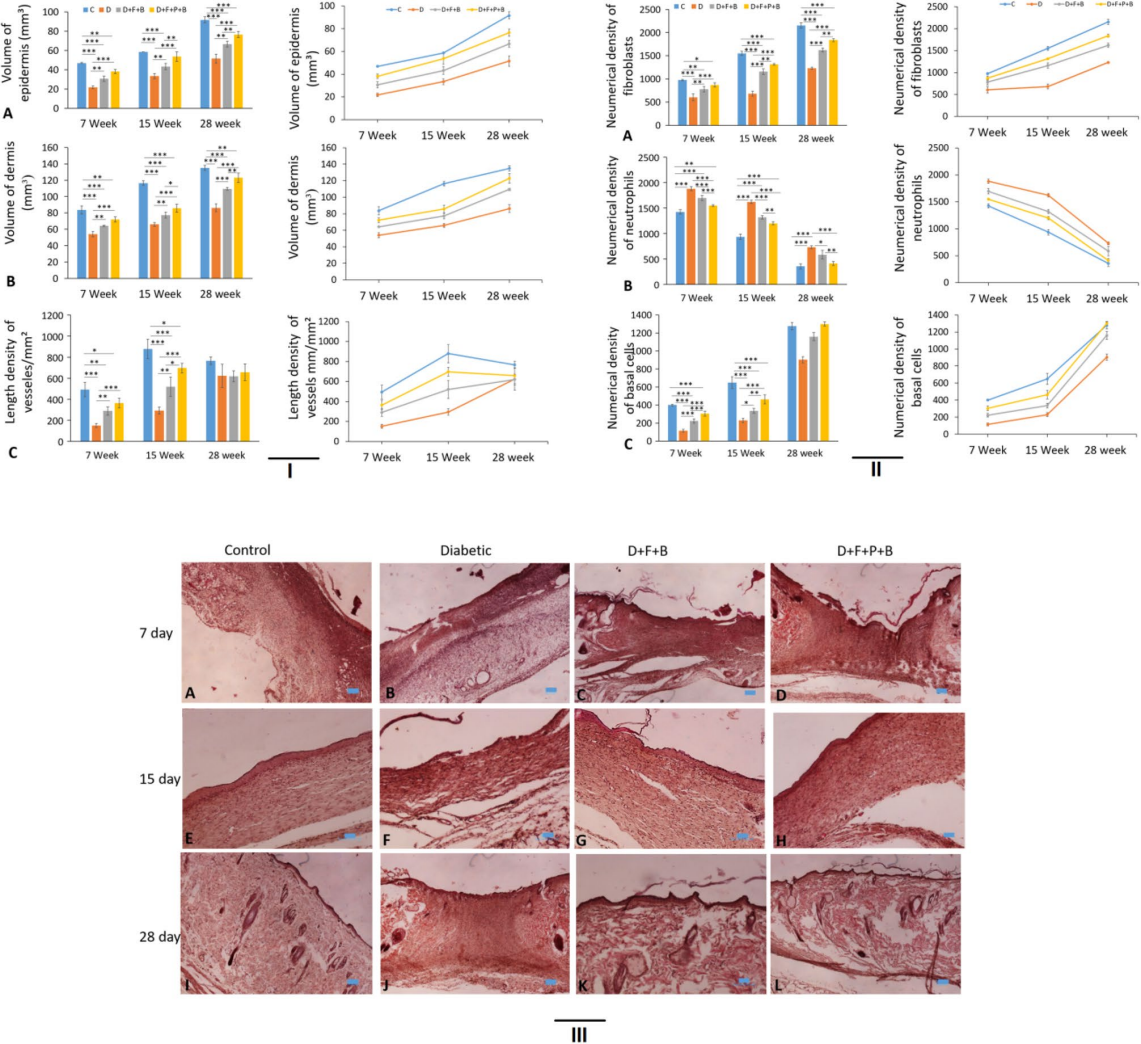
(**I**) Stereological analysis of wound tissue at different time points post-injury. (A–C) Mean ± SD of the total volume of epidermis and dermis at days 7, 5 and 28 post-injury in the wound healing site in the study groups as compared by the one-way ANOVA and LSD; (*P < 0.05, **P < 0.01 and ***P < 0.001). (**II**) Stereological analysis of wound tissue at different time points post-injury. (A–C) Mean ± SD of the number of fibroblasts, neutrophils and basal cells at days 7, 5 and 28 post injury in the wound healing site in the study groups as compared by the ANOVA and LSD; (*P < 0.05, **P < 0.01 and ***P < 0.001) and Repeated Measures. ANOVA, analysis of variance and LSD, least significant difference; C; D; D + F + B; D + F + B + P. (**III**, A–L) Micrographs of a wound healing area following injury in different groups at 7, 15, and 28 days in the study groups. H&E staining. Scale bar = 10 µm.

The wound closure area was determined by using a digital camera, for imaging, and the ImageJ software. The wound area results showed that there was no significant difference in the surface area of wound healing at day 1 post-injury. According to Fig. 8, from the day 7 post-injury, the process of wound healing in group D + F + B + P has occurred in a sharper slope in comparison to the other groups. From days 7, 15, and 28, wounds of the diabetic groups showed a decreased capacity to heal and remained progressively larger until day 28 compared to the other groups. Wound contraction, at days 7, 15, and 28 post-wound induction, showed the significantly enhanced wound closure of treatment groups by D + F + B and D + F + B + P (P < 0.05 and P < 0.01), respectively (Figs. 6 and 7).

### Volume of dermis and epidermis

In Fig. 8IA,IB, the volume of dermis and epidermis in all samples has been increasing in three spans of time (7, 15, 28 days) which indicates on the wound healing. Based on the data, it can be observed that the volume of the dermis and epidermis of the diabetic group (D) has had the most reduction in comparison to that of the Control group, whereas the diabetic group receiving the wound dressing (D + F + B + P), compared to the Control group, shows the least difference (Fig. 8III).

**Figure 9:**
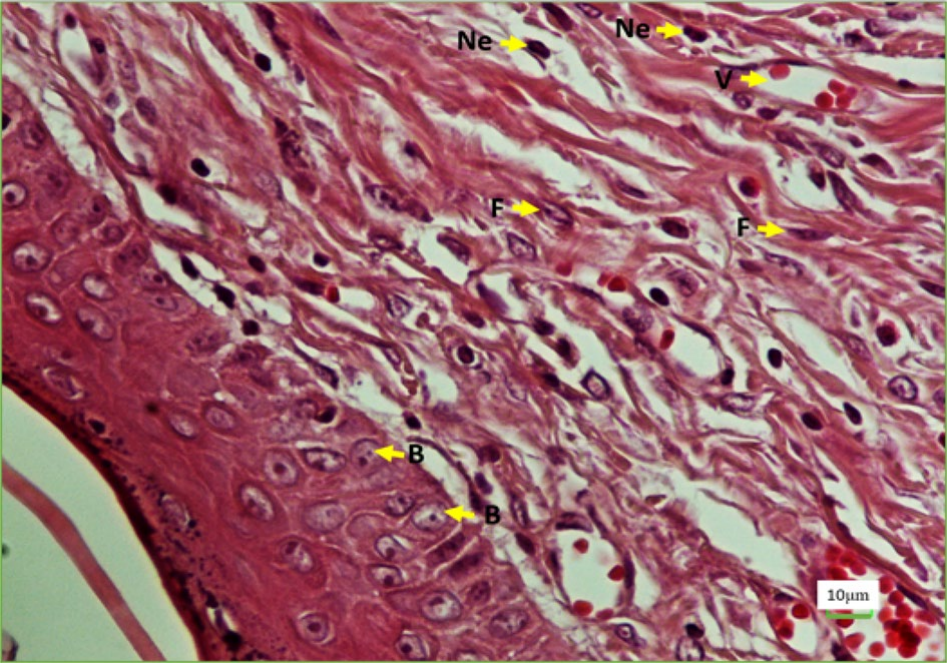
Photomicrograph of the wound healing area following injury stained with H&E (×40). *Ne* neutrophils, *F* fibroblasts, *B* basal cells, *V* vessel.

Figure 8IC reveals the compression of the changes along the vessels during the period of 28 days of wound healing. During 7 and 15 days after surgery, this rate of compression in groups D and D + F + B is approximately half of that in the Control group. These two groups on the 28th day after creating the wound have had remarkable growth; however, group D + F + B + P has experienced completely diverse procedure and has had less decrease compared to the Control group (Fig. 9). Also, on the 15th day, it was increasing while on the 28th day, it was nearly stable.

### Stereology

In all surveys, the D + F + B + P group has presented better results than D and D + F + B groups. According to these figures, the Control group has had better results (Fig. 8II).

On the 7th day:

The level of fibroblasts in all groups in relation to the Control group has decreased. However, according to Fig. 8II, it can be found that the reduction in group D + F + B + P has been much less than that in groups D and D + F + B. The survey of the surface of neutrophils reveals that there has been a considerable increase in all groups compared to that in the Control group, while group D has had the most increase. The survey of the number of Basal cells shows that all three groups have experienced a decrease, compared to the Control group, while group D has had the most decrease among the groups.

On the 15th day:

In the survey of the fibroblasts, it is observed that the surface of fibroblasts in all groups has increased on the seventh day. Group D + F + B + P has experienced the highest increase in comparison to the Control group on this day. The number of neutrophils also has decreased in all groups. In the meantime, the number of neutrophils of the group D + F + B + P has the highest reduction compared to that of the other groups. The increase in Basal cells is observed in all groups, As before, the samples in group D + F + B + P, compared to the Control group, have had a considerable increase among the other groups.

On the 28th day:

Similar to the case of the 15th day after surgery, the number of fibroblasts in samples of the D + F + B + P group, have increased considerably in comparison with that in other groups. Furthermore, the level of neutrophils has been decreasing during this period of time, while the number of neutrophils in samples of the D + F + B + P group and Control group has reached close together. It should be remarked that there has been a considerable increase in Basal cells in a similar period of time in a way that their number in samples of the D + F + B + P group has been more than that in the samples of the Control group. At the same time, the number of these cells in group D varies a lot from that of in the Control group.

### Tensiometery

**Figure 10:**
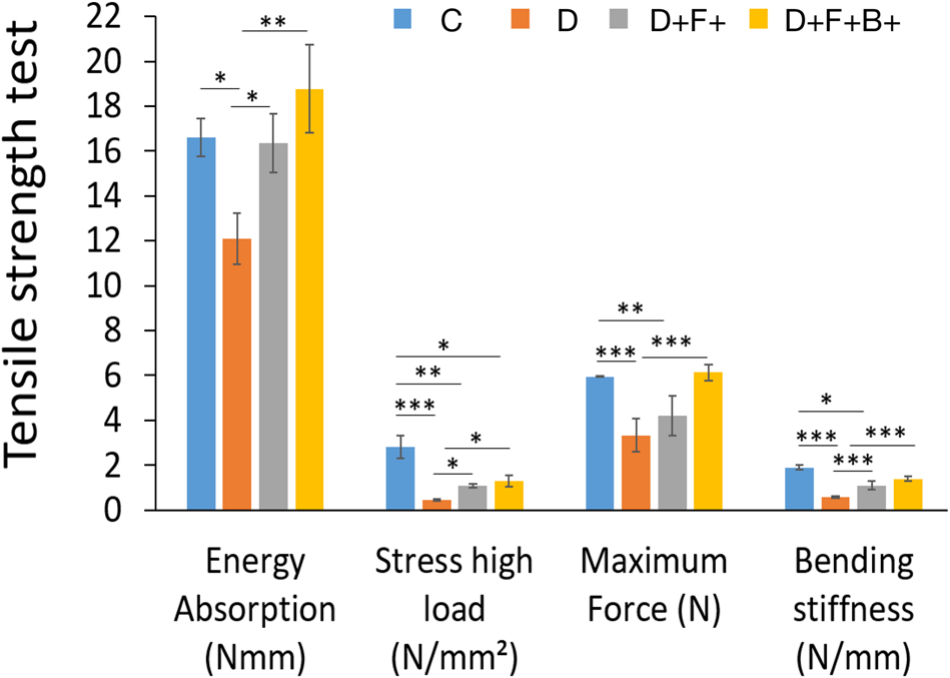
Tensiometery analysis of wound tissue at different time points post-injury. Mean ± SD of the energy absorption, wound strength, bending stiffness, maximum force and stress high load at 28 post-injury in the wound healing site in the study groups as compared by the ANOVA and LSD; (*P < 0.05, **P < 0.01 and ***P < 0.001). One-way ANOVA, analysis of variance and LSD, least significant difference; C; D; D + F + B; D + F + B + P.

The results of tensile strength are shown in Fig. 10 From the figures related to the survey of Bending Stiffness and Stress High Load, it can be seen that the samples of the D group have a significant decrease respect to that of the Control group, whereas the amounts related to the D + F + B + P group, compared to the Control group, have only a little reduction.

From the figure related to the survey of Maximum Force, it can be seen that this parameter in the samples of both groups D and D + F + B, compared to that in the Control group, have decreased significantly; on the other hand, the amounts of this parameter for the samples of the D + F + B + P group have been a bit more than those of the Control group.

Studying figures related to Energy Absorption reveals that a significant increase in the quantities of this parameter has been observed for the samples of the D + F + B + P group, compared to those of the Control group, while these quantities for the samples of group D have considerably reduced.

### Gene expressions

**Figure 11:**
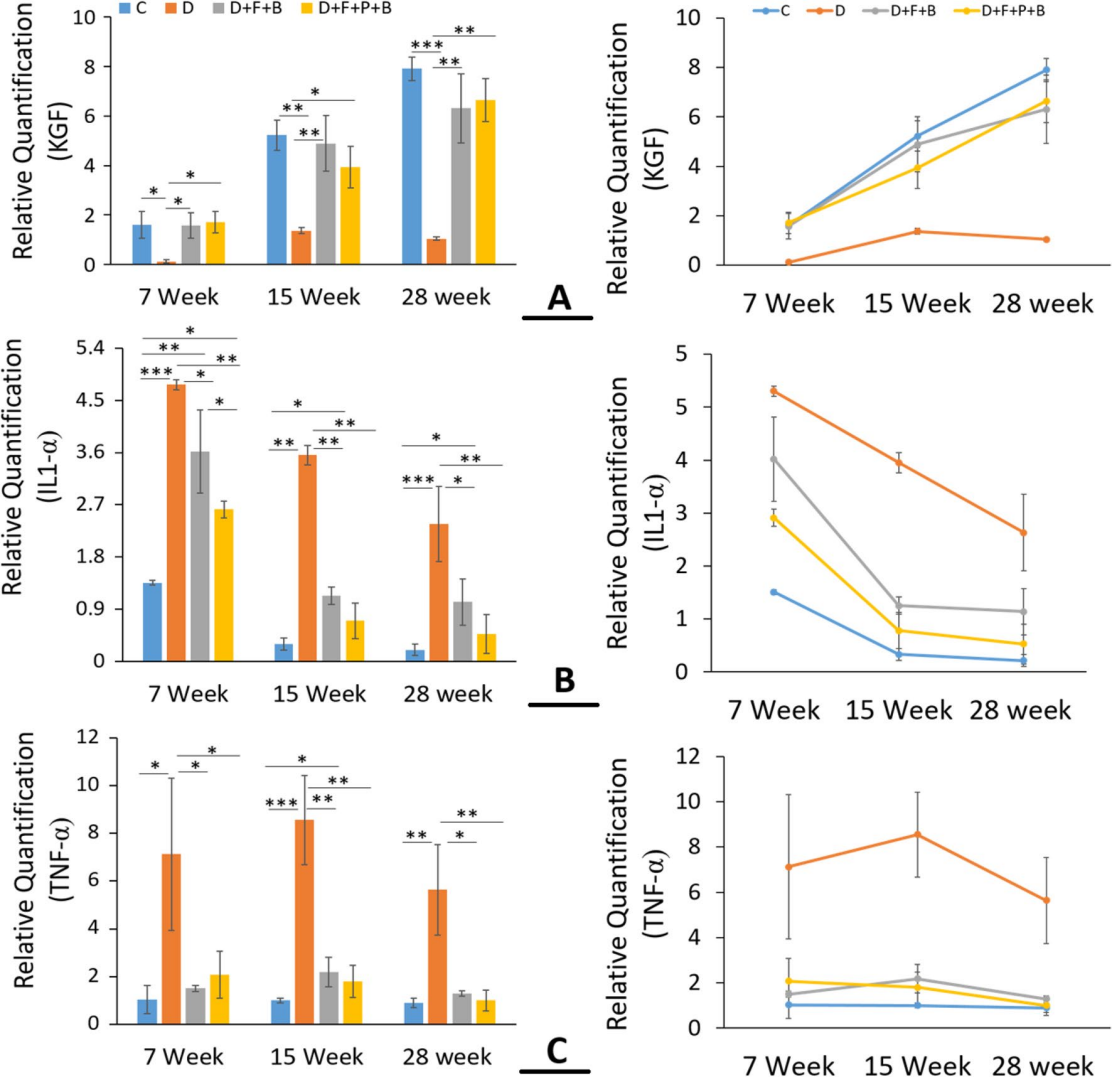
Molecular analysis of wound tissue at different time points post-injury. (**A**–**C**) Mean ± SD of the levels of mRNA expression in KGF, IL1-α and TNF-α at days 7, 5 and 28 post-injury in the wound healing site in the study groups as compared by the ANOVA and LSD; (*P < 0.05, **P < 0.01 and ***P < 0.001) and Repeated measures ANOVA. ANOVA, analysis of variance and LSD, least significant difference; C; D; D + F + B; D + F + B + P.

Figure 11A presents the gene expression of KGF of group D + F + B + P in 7 and 15 days and 28 days after performing the surgery and entering to the phase of improving the wound. A similar behavior is observed for the Control group. Indeed, the results for these two groups are more desirable.

Figure 11B,C show that the gene expression of IL1-α and TNF-α in group D + F + B + P in all three time intervals, after passing the inflammation phase, has a better trend than that in groups D and D + F + B.

## Discussion

Based on the recently published statistics, nearly 8.5% of the population of the world consisting of about 422 million people is suffering from diabetes mellitus^49^. Approximately 15% of the diabetic population develop a foot ulcer and 14–24% of this group of patients were forced to have amputations^50^.

A DDS is described as a platform to load active compounds, enhance the safety and efficacy of the drug, and release them in the target tissue^51^. The main purpose of a DDS is to keep the drugs at optimal therapeutic levels in the body during the treatment, so multiple administrations can be prevented. In this scenario, the implants of polymer become ideal for loading. Throughout the time of the therapy, one or more active compounds are released at the desired release rate. Also, scaffolds provide drug protection in the body before release. It is also important to prevent side effects during the controlled release and fluctuations of drug levels from multiple sequential administrations of immediate drug releases. It is even more relevant as the drug content increases. Since scaffolds are implanted on a site-specific basis, a more controlled spatiotemporal release of active compounds can be obtained when compared to conventional formulations^52,53^. In addition to drugs, in order to release cells, proteins, and genes, biocompatible scaffolds are currently used^54,55^. These applications enhance their limits for the designing of novel treatments for diseases. These include tumor treatments and pathogen-derived microbial, and the stimulation of biological responses in regenerative medicine as a device of tissue repair^56^.

Nowadays, knitted polypropylene is the material that is most frequently used to repair a ventral hernia. The sturdy mechanical properties of polypropylene and its beneficial effect on the ventral hernia repair and the prolapse of the pelvic organ are precisely documented^24^. To reconstruct a variety of tissues including the skin^57^, body wall^58,59^, urinary bladder, and rotator cuff muscle among others^60^, biologic scaffolds consisting of allogeneic or xenogeneic ECM have been employed. Several strategies have been conducted, aiming to minimize the fibrotic response to synthetic mesh materials including alternations in the mesh fiber composition, reduction of mesh surface area, and application of bioactive coatings^24^. The present study reveals that loaded-BET has a clear and distinct long-term effect on the host response to the plasma-treated PP mesh. Cell attachment and proliferation can be provided by polymer scaffolds. The Healing and integration of the tissue are achieved by the physical bonds between biological parts and scaffolds^61^. When an implant is fixed in a person’s body, the risk of infection increases^62^. Plasma treatment is considered to be one of the sterilizing techniques that can be applied to the materials in order to prevent the implanted place from potential infection^62^. For the purposes of sterilization and sanitizing, activation with plasma can be used on the biological materials, which may be damaged by other difficult chemical conditions and temperature^63^. Moreover, the irradiation of plasma in a short period of time never causes the materials to be too much warm, so its destruction is prevented. The other distinguished advantages of plasma, turning it into a desirable means of surface modification for various biomedical applications, are treatment speed, the deprivation of the need for solvents, and practical measurability^64–66^. In the previous decade, this approach enjoyed more popularity among drug applications, as well^19,67–73^. The major factor that indicates the drug release rate is the degree and the level of materials’ hydrophobicity^29^. So the increase in surface wettability of the polymer can be created with the introduction of new functional groups^9,32,74^, or surface roughness^16,75,76^. According to the studies conducted, plasma treatment, especially with oxygen gas, has had a remarkable effect on the increase in hydrophilicity rate^10,12,16,77^, and also on the roughness of PP surface^10,77,78^.

Recent discoveries suggest that polymer-based drug delivery scaffolds release various factors that are important in the cellular normal physiological function during the culturing process^56^.

In order to manufacture wound dressing that has the capability of drug release, both plasma Functionalization, and plasma polymerization, two different applications of plasma, have been exploited. Meanwhile, by using plasma, two stages of setting drug release can be precisely fixed and the delayed-release can be achieved^79^.

The analysis of the results of the ATR-FTIR test shows that in the sample treated with plasma, in comparison with the Control sample, there are vibrations with the wave number related to the group of hydroxyl and carbonyl (3459 cm^−1^ and 1638 cm^−1^). Being exposed to plasma has caused the new polar functional groups including carbonyl, hydroxyl, and etc. to activate the polymer surface. Therefore, the creation of these functional groups enhances the polymer-free surface energy considerably^16,80–83^. Consequently, the plasma treatment can be used as a helpful means of increasing the amount of drug loaded^16^.

In order to estimate the components on PP mesh, before and after plasma treatment, energy dispersive X-ray microanalysis (EDX) were carried out. Before plasma treatment, the only element existing on the PP mesh was carbon, while after activating the surface of the mesh and loading BET, besides carbon, elements such as oxygen, nitrogen, and chlorine appeared. Each of these three elements is observed in the chemical structure of the drug, whose presence in spectra indicates on the physical bond of the drug with mesh surface. Meanwhile, a proportion of carbon and oxygen observed can be because of the appearance of functional groups resulted from the treatment with oxygen plasma. An increase in the percentage of the atoms of nitrogen, oxygen, and chlorine on the surface of the F + B + P sample, in comparison with the F + B sample, signifies that more drug loaded on the wound dressing can lead to an effective release of the drug, and consequently wound improvement.

Generally, untreated pp surface is a completely smooth surface^84,85^. By using the glow discharge plasma with oxygen gas, the surface roughness has been remarkably changed and plasma has done the etching. According to the previous studies^86–88^, plasma treatment of the surface of the amorphous polymer is a complicated process including combined etching, photo-oxidation, and thermal oxidation^45^. By analyzing the pictures taken from FESEM we can conclude that the most amount of drug loaded is related to the samples functionalized. By using plasma polymerization, monomer tetraglyme is used to create the PEG-like layer on PP on which the polymeric network can be easily seen.

To achieve the HPLC peak of BET, a variety of methods, among the existing and common methods, were tested^36,89^. But during the experiments, no peak was observed. By changing the fore-mentioned approaches which are adjustable to the machine’s conditions, finally, by adjusting the machine to the conditions mentioned in “High-performance liquid chromatography method (analysis) (HPLC)”, the peak related to the BET was obtained. Then, we thoroughly examined the range of the loaded and the released drug and the effect of the consumption power and the duration of the application of plasma on the mesh. Study the application time of the glow discharge plasma with oxygen gas showed that the increase in power consumption is inversely related to the amount of the drug loaded. Besides, the results show that an increase in power consumption causes the release of 10% of the loaded drug. When 50 W power is used, the released drug is shown to be less than 10%.

The analysis of the effect of plasma application time reveals that the amount of the drug loaded, depending on the time of surface treatment, has increased in the samples treated with both 50- and 70-W consumption powers; on the other hand, the amount of the drug released has had a completely opposite behavior which shows that the amount of the drug released has decreased due to the increase in time of plasma irradiation. Thus, analyzing the results reveals that an acceptable amount of drug loading and release is obtained under the condition of 70 W of consumption power and irradiation of 15 min.

In order to avoid aging^90^, the process of drug loading on the PP mesh was done immediately after the application of plasma for the purpose of functionalization. After the distinction of the conditions of the first stage of plasma and drug loading, plasma polymerization was applied on samples. The PEG-like coating that is obtained by the sedimentation of tetra-ethylene glycol dimethyl ether (tetraglyme) using plasma treatment can deliver the antifouling property to the polymeric surface^91,92^. Furthermore, it was found out that the cross-linking creates the free radicals which in turn leads to polymerization of collagen fibers and boosts the corneal tissue^93^. Therefore, here it is considered that cross-linking obtained from the plasma polymerization would be useful for the polymerization of collagen fibers and the reinforcement of skin tissue.

On the other hand, two periods of time, 15 and 30 min, for plasma polymerization and also, utilization of Argon gas as a carrier and diluting gas were studied. It was observed that PEG2 and PEG3 show the most similar figures to the figure of zero-order controlled release^46^. However, it was pointed out that the increase in the time period of plasma polymerization can cause the abortion of the drug release^14^. The comparison of the results obtained from these two samples signifies that the percentage of the drug released in the PEG2 sample is a bit more than that in the PEG3 sample. Hence, the study of the process of drug release in the sample on which the plasma polymerization has been applied for 15 min using argon gas at a flow rate of 2 sccm as a carrier gas and also at a flow rate of 8 sccm as a diluting gas shows that after 240 h of sample immersion in PBS solution, around 30% of the existing drug has released on its surface. The analysis of the release procedure indicates that the drug release has occurred regularly and continuously in a specific period of time and could influence the procedure of wound improvement.

The procedure of drug release in the F + B and F + B + P samples was studied. The procedure in both samples was nearly similar to zero-order controlled release^46^. This similarity was seen in most tests related to the drug release. Besides, it was observed that the impact of F + B + P sample on the improvement of rats’ wound was more than that of F + B sample.

In the present study, stereo-logical techniques have been employed in order to have unbiased accurate estimations. This study revealed that applying F + B + P boosts fibroblast and basal cell proliferation and vascularization. Moreover it manifested anti-inflammatory effects on epithelialization with thick dermis occurs in the F + B + P group. This study displayed that there is a meaningful increase the healing process of skin wounds. Besides, the results stereo-logically obtained suggest that in the length of vessels and wound closure area compared to the other groups in days 15 and 28.

The healing of wounds is divided into three phases of inflammation, proliferation, and remodeling. The inflammatory cells play a vital role in the inflammation phase until the induction of fibroblast proliferation and the formation of new blood vessels^94^.

Our results showed that the number of neutrophils in the F + B + P group is significantly lower than that in the other groups. This could be due to several factors of betaine, scaffold, and plasma (F + B + P), which may resolve and shorten the inflammatory phase and induce angiogenesis. In addition, neutrophils produce TNF-α and IL-1 which recruit fibroblasts and epithelial cells^95^. Macrophages enter the site of injury and take part in the phagocytic process by releasing growth factors and cytokines: PDGF, TGF-ß, ß-FGF, TNF-α, IL-1, and IL-6, that help bring in the proliferative phase of healing^96^. Epithelialization proceeds with proliferation and migration of the epithelial cells with the aid of EGF, keratinocyte growth factors (KGFs), and TGF-α^97^.

Betaine possesses various pharmacological activities, such as anti-inflammation, anti-fibrosis, and anti-oxidation^98^. It was suggested that the BET can decrease the levels of serum TNF-α, which agrees with our results^99^. Furthermore, consistent to our results, the western blot results of BET treatment was reported which suggests that this treatment down-regulates the protein expression of TNF-α and IL-1β significantly^98^. Moreover, from a series of studies it was concluded that BET could up-regulate insulin-like growth factor 1(IGF-1) gene expression in rat liver^100^, and IGF-1 could up-regulate β-defensin gene expression in keratinocytes of humans^101^. Expectedly, the findings of the present research confirm these reports.

On the other hand, Fibroblast cells are activated in the wound area in the proliferation phase^95^. There is a significant increase in fibroblast cells in the F + B + P group on days 7 and 15 of the study, which indicates the earlier initiation of the proliferation phase in the F + B + P group. These results provide strong evidence that some factors in the F + B + P group may activate fibroblasts and cell proliferation.

In our non-diabetic and diabetic rats with wound dressing, healed wounds displayed a significant increase in maximum force, stress high load, and energy absorption on the 28th-day post-injury compared to those of the D group. Our biomechanical analysis revealed that the healing of wounds in rats that received F + B + P mesh was significantly stronger (maximum force) than that of the D group.

It should be noted that the groups of control and F + B + P show higher bending stiffness because increased fibrillar molecules per unit area increases fibril diameter or cross-linking of fibrillar molecules^.^^102^.

## Conclusion

In this study, two different plasma processes (Plasma Functionalization and Plasma Polymerization) are employed in an innovative method to design PP meshes for the controlled drug release on the Diabetic wounds. Plasma functionalization at low-pressure has been applied to modify PP fiber surfaces at a nano-metric level. This modification is achieved by functionalizing the surface with polar oxygen groups. These are related to a progressive increase of wettability as well as surface roughness which is dependent on the exposure time. The chemical and morphological changes produced on the surface of PP fibers provide a better availability of bonding sites for subsequent attachment of molecules and are related to higher BET loaded in the PP meshes after 15 min of plasma treatment. However, these treatments alone are associated with altered morphology. Thus, the plasma-functionalized PP meshes containing high antioxidant loads gain a polyethylene glycol-like layer (F + B + P) that allows higher BET loadings. Specifically, the F + B + P mesh decreases the inflammation and shortens the wound healing course of diabetic rats. These findings are promising and may lead to improved clinical consequences in other regenerative medicine and biomaterial applications.

## Data Availability

All relevant data are available within the paper.
